# Time-Specific Pattern of Iron Deposition in Different Regions in Parkinson's Disease Measured by Quantitative Susceptibility Mapping

**DOI:** 10.3389/fneur.2021.631210

**Published:** 2021-08-04

**Authors:** Xiaodi Fu, Wenbin Deng, Xiangqin Cui, Xiao Zhou, Weizheng Song, Mengqiu Pan, Xiao Chi, Jinghui Xu, Ying Jiang, Qun Wang, Yunqi Xu

**Affiliations:** ^1^Department of Neurology, Nanfang Hospital, Southern Medical University, Guangzhou, China; ^2^Department of Neurology, Johns Hopkins University School of Medicine, Baltimore, MD, United States; ^3^Department of Neurosurgery, the Eighth People's Hospital of Chengdu, Chengdu, China; ^4^Department of Neurology, Guangdong 999 Brain Hospital, Guangzhou, China; ^5^The Third Affiliated Hospital, Sun Yat-sen University, Guangzhou, China

**Keywords:** quantitative susceptibility mapping, parkinson's disease, iron deposition, unified parkinson's disease rating scale, hoehn-yahr stage

## Abstract

Studies have shown the spatial specificity of cranial iron deposition in different regions in Parkinson's disease (PD). However, the time-specific patterns of iron deposition are not yet clear. The purpose of this study was to investigate the time pattern of iron variations and its clinical relevance in multiple gray matter nuclei in PD using quantitative susceptibility mapping (QSM). Thirty controls and 33 PD patients were enrolled, namely, 11 cases of early stage of PD (ESP) and 22 cases of advanced stage of PD (ASP) according to the Hoehn-Yahr stages. The iron content in the subcortical nuclei covering substantia nigra (SN), red nucleus (RN), head of the caudate nucleus (CN), globus pallidus (GP), and putamen (PT) was measured using QSM, and the clinical symptoms of PD were evaluated by various rating scales. The QSM values in SN, RN, GP, and PT significantly increased in PD patients compared with the controls. Further subgroup comparison with the controls indicated that the iron content in SN and GP (paleostriatum) gradually elevated in the whole disease duration and was related to clinical features. While the iron content in RN and PT (neostriatum) only elevated significantly in ESP patients, further iron deposition was not obvious in ASP patients. Our study confirmed that QSM could be used as a disease biomarker and could be suitable for longitudinal monitoring. However, considering the temporal characteristics of iron deposition in neostriatum, iron deposition in the neostriatum should be paid more attention in the early stage of the disease, even in the preclinical stage, in future research.

## Key Points

- The iron deposition of Parkinson's disease showed the regional specificity of distribution, especially in the substantia nigra.
- The iron deposition of Parkinson's disease also showed the time-specific features. The progress of iron deposition in different brain regions is heterogeneous.
- The QSM values could be used as a useful disease biomarker and be suitable for longitudinal monitoring in PD. QSM values in neostriatum should be paid more attention in the early stage of the disease, even in the preclinical stage.
- These characteristics of iron deposition help us to understand the pathological changes of Parkinson's disease.

## Introduction

Parkinson's disease (PD) is the second most common neurodegenerative disease and commonly manifested by typical motor symptoms such as bradykinesia, resting tremor, rigidity, postural instability, and an array of non-motor symptoms, which seriously affect the quality of life and exacerbate the disability of PD patients. The major pathology features of PD are characterized by the loss of dopaminergic neurons in the substantia nigra (SN), the accumulation of Lewy bodies and Lewy neurites (1), and excessive iron deposition in specific brain regions (2). Studies have shown that iron deposition in the SN had a pivotal role in the necrosis of dopaminergic neurons (3) and the aggregation of α-synuclein (4, 5) and promoted the progress of the disease (6).

Previous studies have confirmed that the overloaded cranial iron was mainly located in the SN, and the iron deposition of SN showed a progressive increase with the progress of PD as well (7–9). The presence of excessive iron has also been found in other subcortical gray matter nuclei such as red nucleus (RN) and globus pallidus (GP) (10). According to Braak's “dual hit theory”, the possible access of the pathogenic progression of Lewy bodies originated from the intestine and nose, respectively, and then gradually spread to the central nervous system *via* two routes (11). However, considering that PD is a highly heterogeneous disease, it is not clear whether the cranial iron deposition in different regions had a similar time sequence.

In this study, we utilized quantitative susceptibility mapping (QSM), which has been validated to be the most accurate method so far for quantitatively detecting cranial iron content (12–15), to measure the iron deposition in different regions of PD brains (16), aiming to explore the spatial and temporal pattern of iron deposition and its relationship with clinical features. This study helped us to deepen the understanding of brain iron deposition in PD patients.

## Method

### Subjects and Agreements

PD patients: A total of 33 PD patients were consecutively recruited from Nanfang Hospital, The Third Affiliated Hospital of Sun Yat-sen University, and Guangdong 999 Brain Hospital. Diagnosis of PD was based on the MDS clinical diagnostic criteria for PD (17) evaluated by two experienced neurologists specializing in neurodegenerative diseases. The demographics of the included subjects are shown in Table 1.

**Table 1:** Demographics of patients with PD and control group.

	**CG**	**PD**	**PD**		***p*_**1**_**	***p*_**2**_**
			**ESP**	**ASP**		
*N*	30	33	11	22	/	/
M/F, *n*	17/13	20/13	8/3	12/10	0.802^a^	0.035 ^*^, ^a^
Age, years	55.6 ± 11.83	60.73 ± 9.57	57.73 ± 8.33	62.23 ± 9.98	0.062^b^	0.178^b^
Duration, years	/	6.25 ± 3.80	4.14 ± 3.21	7.30 ± 3.70	/	0.022 ^*^, ^b^
Hoehn-Yahr stages	/	2.55 ± 0.52	1.96 ± 0.15	2.84 ± 0.36	/	<0.001^**^, ^b^
UPDRS-III	/	48.76 ± 17.04	33.73 ± 13.56	56.27 ± 13.32	/	<0.001^**^, ^b^
MMSE	/	25.55 ± 3.61	26.73 ± 2.53	24.95 ± 3.97	/	0.189^c^
HAMA	/	13.00 ± 8.31	12.18 ± 9.79	13.41 ± 7.69	/	0.696^c^
HAMD	/	18.15 ± 8.97	14.64 ± 11.30	19.91 ± 7.22	/	0.113^b^
Medication (yes/no)	/	27/6	8/3	19/3	/	0.375^a^

*Data are presented with means ± standard deviations. p_1_, CG vs. PD; p_2_, ESP vs. ASP. N, (numbers); M, (male); F, (female); ESP, (early stage of PD); ASP, (advanced stage of PD); UPDRS-III, (Unified Parkinson's Disease Rating Scale III); MNMSS, (Main Non-motor Symptoms Scores); MMSE, (Mini-mental State Examination); HAMA, (Hamilton Depression Scale); HAMD, (Hamilton Anxiety Scale)*.

a *Pearson chi-square test*.

b *Independent two-tailed t-test*.

c *Mann-Whitney U test*.

* *p < 0.05*,

** *p < 0.01*.

Subjects in the control group: A total of 30 age- and gender-matched control patients were enrolled in this study. The subjects in the control group were from outpatient clinics in Nanfang Hospital who mostly complained of dizziness, headache, or other mild neurological diseases. The specific exclusion criteria are as follows: (1) severe neurological symptoms and positive signs; (2) known history of severe neurological disorders, such as cerebrovascular disease, encephalitis, brain tumor, meningitis, extrapyramidal disorder, and other intracranial lesions; (3) head trauma and history of surgery; and (4) mental illnesses or psychological diseases.

The study was approved by the Ethics Committee of Nanfang Hospital, and all subjects enrolled in this study provided written informed consent before participation, and all experiments were performed in accordance with the relevant regulations.

### Clinical Assessments of PD Patients

All patients with PD were assessed by a series of rating scales for evaluating the motor and non-motor symptoms. Severity of PD was assessed by H-Y stage (18), and the third part of the MDS-Unified PD Rating Scale score (UPDRS III) (19) was used to measure the severity of motor symptoms. Non-motor symptoms were assessed by a series of clinical rating scales, including the Mini-Mental Status Examination (MMSE) for cognitive impairment, and the Hamilton Anxiety Scale (HAMA) and the Hamilton Depression Scale (HAMD) for anxiety and depression, respectively. All assessments of scales were evaluated by two experienced neurologists who were trained in the use of clinical neuropsychological scales.

PD patients were subdivided into two subgroups according to H-Y stage. Eleven cases with H-Y stage <2.5 were classified as the early-stage PD (ESP) subgroup and 22 cases with H-Y stage ≥2.5 were the advanced-stage PD (ASP) subgroup. All PD patients were evaluated after withdrawing all antiparkinsonian medicine for at least 4 h.

### MRI Examination

All MR images were acquired using a 3.0-T MRI system (Magnetom Trio Tim; Siemens Medical Solutions, Erlangen, Germany) with an eight-channel head coil. Imaging parameters for the three-dimensional, multi-echo gradient echo sequence were as follows: TE of first echo = 5.9 ms, echo spacing = 7.0 ms, number of echoes = 5, TR = 38 ms; matrix = 183 × 156; field of view = 182 × 220 mm^2^, slice gap = 0.6 mm, slice thickness = 2 mm; flip angle = 10°. All subjects used a fixed foam pad to restrict the head movement during MRI scanning for a better image quality.

### Image Post-processing and QSM Reconstruction

The acquired 3D multi-echo GRE data were used for reconstructing QSM imaging by the STI Suite software (20) (Duke University). Firstly, we obtained the total field map from the original MRI data, and original phase images were unwrapped based on Laplacian algorithms (14). Due to the background fields hindering the tissue phase contrast in the unwrapped phase, further removal of the background phase was necessary using the Sophisticated harmonic artifact reduction for phase data (SHARP) (21). Ultimately, the inversion calculation of tissue fields was performed by the streaking artifact reduction for QSM (STAR-QSM) (22) before obtaining the high-quality images. As the intensity of every voxel reflects the relative susceptibility, the average susceptibility value of cerebral–spinal fluid in the anterior horns of the ventricles was used as a reference value to normalize the QSM maps (23, 24).

### Measurement of Regions of Interest (ROIs)

**Figure 1:**
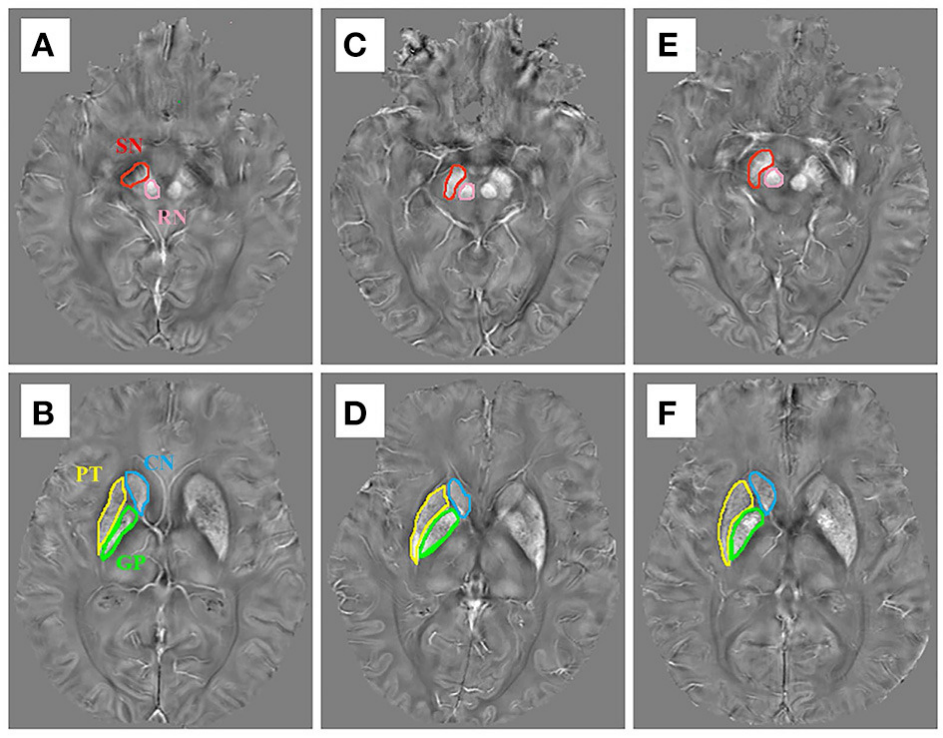
The susceptibility maps illustrate the five regions of interest in **(A,B)** a control case, **(C,D)** an early stage of PD case, and **(E,F)** a late-stage of PD case.

In this study, ROIs of the following areas were obtained from both sides of the brain: SN, RN, CN, GP, and PT. These five ROIs were manually delineated on the QSM images by the ITK-SNAP software (Figure 1). First, we opened the QSM images in the ITK-SNAP software and adjusted the contrast of the acquired images, then selected the clearest layer and two adjacent layers of each nucleus and performed manual segmentation. For example, the first layer of SN was started either at the level of the RN showing the largest diameters or the next slice, depending on the layer where the SN displayed most prominently, and then a total of three layers were used (25). Finally, further calculation was performed by the software to obtain the one-sided average magnetic susceptibility of the three layers and then the bilateral mean values were used as the regional QSM values for further analysis. All steps were performed by two experienced neurologists who were blinded to the diagnosis of each subject.

### Data Analysis

All data analyzed in this study used the standard statistical package SPSS V. 20.0 (IBM Corporation, Armonk, NY), and the graphical depictions were made using GraphPad Prism v. 6.07 (GraphPad Software, Inc. La Jolla, CA, USA). All the continuous variables, including age, disease duration, scores of clinical scales, and regional QSM values, were expressed as means ± standard deviations. The single-sample Kolmogorov–Smirnov test was performed on all continuous data, and normal distribution was confirmed if *p* > 0.05. In this study, except for the HAMA and MMSE scores of PD group, all other variables were normally distributed.

Discrete variables such as gender comparisons between control and PD groups were performed by Pearson χ^2^ test. For the continuous variables, if normally distributed, a two-tailed *t*-test was used for the comparison. If not normally distributed, variables were assessed by Mann-Whitney *U* test. One-way analysis of variance (ANOVA) was used to compare the differences in QSM values of different ROIs among the subgroups, and the least significance difference (LSD) method was used for post-*hoc* analysis, and the *p*-value was adjusted by Bonferroni correction. As the physiological iron accumulation may increase with age in the normal population, covariance analysis was used to investigate the differences of QSM values between control cases and each subgroup of PD cases, respectively. Pearson correlation analysis was conducted to explore the relationship between the iron deposition of each ROIs and the clinical symptoms of PD patients (parameters involved were in normal distribution). Spearman correlation analysis was conducted to explore the relationship between the iron deposition of each ROIs and MMSE or HAMA. The receiver operative characteristic (ROC) analysis was performed to evaluate the diagnostic value of potential biomarkers in all PD.

Statistical significance was defined by a *p*-value < 0.05, which was indicated by ^*^; if the *p*-value was < 0.01, data were indicated by ^**^. The corrected *p*-value in multiple comparison was < 0.025.

## Results

### Demographic and Clinical Data of Enrolled Subjects

Table 1 reports the demographic characteristics of all participants. A total of 63 cases were enrolled in the study, namely, 33 Parkinson's disease patients and 30 controls. No statistical difference was found in gender and age between the PD group and the control group (CG).

### Comparison of QSM Values in Different Brain ROIs Between PD Groups and Control Groups

**Figure 2:**
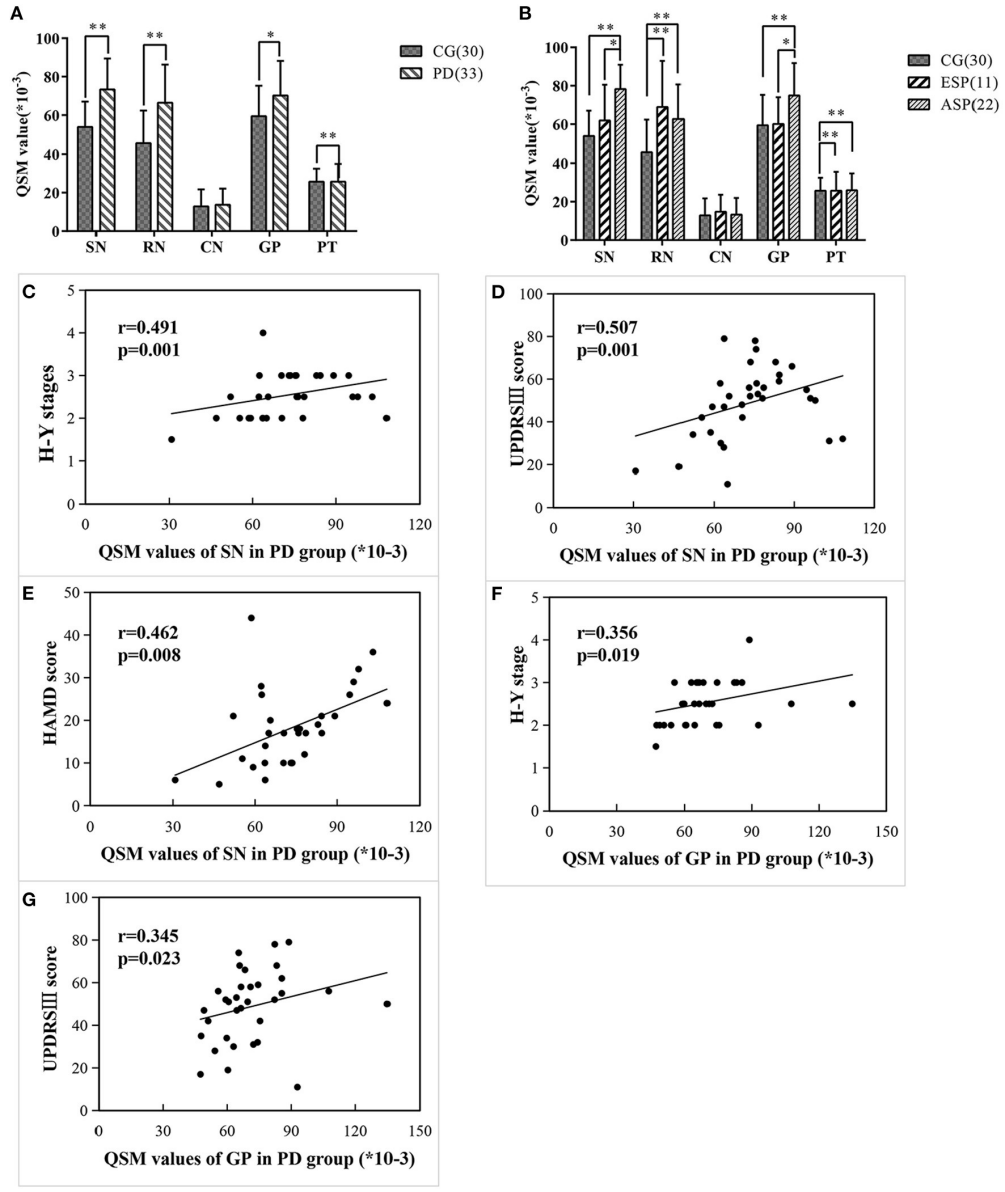
Comparisons of regional QSM values between different groups/subgroups and the correlations between regional QSM values and clinical data in PD cases. **(A,B)** Differences of regional QSM values between the CG and PD group **(A)** and CG and two subgroups **(B)** using covariance analysis adjusted for age. Significant differences are represented as: *p < 0.05, **p < 0.01. **(C–E)** QSM values of SN significantly correlated with H-Y stage **(C)**, UPDRSIII score **(D)**, and HAMD score **(E)** in PD patients. **(F,G)** QSM values of GP significantly correlated with H-Y stage **(F)**, UPDRSIII score **(G)** in PD patients.

The QSM values of five regions of two groups are summarized in Table 2, Figure 2A. Covariance analysis was used to reduce the possible interference on the iron deposition due to the age growth. The QSM values of SN, GP, RN, and PT in the PD group exhibited a significant increase compared with the control group, whereas the QSM values of CN showed no significant difference.

**Table 2:** Comparisons of regional QSM values (×10^−3^) between CG and PD, CG and ESP, and ESP and ASP, respectively.

**(1)**	**CG**	**PD**	**PD**		***p*_**1**_**	***p*_**2**_**	***p*_**3**_**
			**ESP**	**ASP**			
*N*	30	33	11	22	/	/	/
SN	53.84 ± 13.20	73.40 ± 16.09	61.97 ± 18.57	78.27 ± 12.69	<0.001^**^	0.052	0.026^*^
RN	45.81 ± 16.55	66.44 ± 19.81	68.95 ± 24.02	62.70 ± 18.01	<0.001^**^	0.009^**^	0.657
CN	12.89 ± 8.82	13.73 ± 8.41	14.76 ± 8.89	13.29 ± 8.65	0.505	0.643	0.798
GP	59.43 ± 15.89	70.22 ± 18.01	60.12 ± 13.92	74.93 ± 16.89	0.016^*^	0.663	0.027^*^
PT	15.63 ± 6.74	25.73 ± 9.22	25.67 ± 9.84	25.92 ± 8.75	0.004^**^	0.012^*^	0.617
**(2) ROI**	***F***	**Sig**	***t***_**1**_	***t***_**2**_	***t***_**3**_		
	**CG ESP**	**ASP**					
SN	9.235	<0.001^*^	0.176	<0.001^*^	0.015^*^		
RN	4.218	0.022^*^	0.015^*^	0.012^*^	0.757		
CN	0.148	0.813	0.602	0.854	0.679		
GP	5.362	0.027^*^	0.815	0.008^*^	0.016^*^		
PT	5.018	0.011^*^	0.018^*^	0.004^*^	0.843		

*QSM values (×10^−3^) are given in means ± standard deviations. p_1_, PD vs. CG; p_2_, CG vs. ESP; p_3_, ESP vs. ASP. Covariances analysis was used for the comparison.*

* *p < 0.05*,

** *p < 0.01*.

One-way analysis of variance was used for the comparison. Between groups: df_1_ = 2. Within groups: df_2_ = 60 Post-hoc test was performed by LSD t-test. t_1_, CG vs. ESP, t_2_, CG vs. ASP, t_3_, ESP vs. ASP. p-value was adjusted by Bonferroni correction. *t < (0.05/2) = 0.025.

### Intercomparisons of Regional QSM Values Among CG, ESP, and ASP

Covariance analysis and one-way ANOVA was used for comparing regional QSM values among subgroups, respectively, and there is no significant difference between two methods. By comparing each subgroup with the control group concerning the regional iron deposition, Table 2, Figure 2B illustrate that QSM values of SN and GP showed no significant difference in ESP cases as compared with the control group, while in patients with advanced stage, the QSM values of SN and GP significantly increased. Instead, QSM values of RN and PT regions in ESP cases were remarkably higher than those in control cases, yet no significant difference was found between ESP and ASP.

### Correlation Between Regional QSM Values and Clinical Features of PD Patients

Further correlation between regional QSM values and clinical measurements of PD cases is summarized in Figures 2C–G. Results indicated that the QSM values of SN were significantly and positively correlated with Hoehn-Yahr stage, UPDRS-III scores, and HAMD scores (*r* = 0.491, *p* = 0.001; *r* = 0.507, *p* = 0.001; *r* = 0.462, *p* = 0.008, respectively). Similarly, QSM values of GP also had a notable correlation with Hoehn-Yahr stage and UPDRS-III scores (*r* = 0.356, *p* = 0.019; *r* = 0.345, *p* = 0.023, respectively) in patients with PD. No correlation was found between the QSM values of RN and PT with clinical assessments of PD.

### Diagnostic Value of QSM Values in RN and PT in Patients With PD

**Figure 3:**
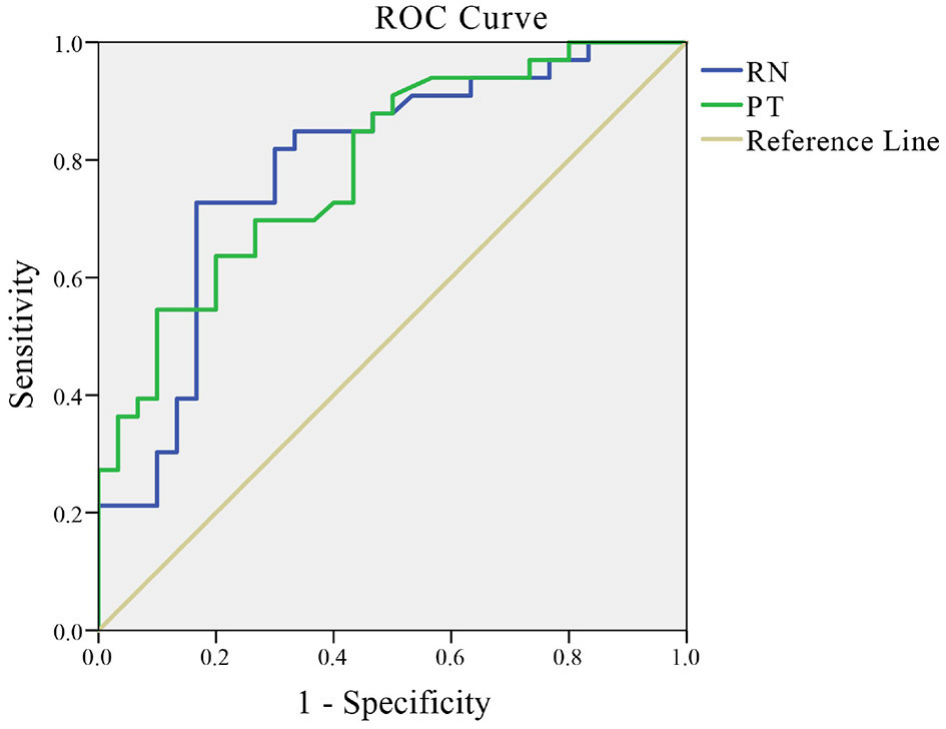
ROC curves of QSM values in RN and PT.

Receiver operating characteristics curve indicated that a cutoff QSM value in RN of 0.0666 resulted in a sensitivity of 72.70% and a specificity of 83.30%, with an area under the curve of 0.788 (95% CI: 0.673–0.904) in order to discriminate PD from control cases (*p* < 0.001). Also, a cutoff QSM value in PT of 0.0377 with a sensitivity of 54.50%, a specificity of 90.00%, and an area under the curve of 0.789 (95% CI: 0.680–0.899) was determined for diagnosis of PD (*p* < 0.001) (Figure 3).

The area under the curve (AUC) of QSM values in RN was 0.788 (*p* < 0.001) (95% CI: 0.673–0.904). Youden's index was maximal with a sensitivity of 72.70% and a specificity of 83.30% when the QSM value in RN was 0.0666. The AUC of QSM values in PT was 0.789 (*p* < 0.001) (95% CI: 0.680–0.899). Youden's index was maximal with a sensitivity of 54.50% and a specificity of 90.00% when the QSM value in PT was 0.0377. ROC = receiver operator characteristic.

## Discussion

In the present study, the significant findings of this study are that iron deposition in Parkinson's disease not only has a region-specific pattern, but also has a time-specific pattern in different regions. Iron deposited in SN and GP increased with the disease duration, and remarkably increased in the late stage of disease. The iron content of RN and PT significantly increased at the early stage of PD but might not continue to increase in the late stage of PD. These characteristics of iron deposition further confirmed that Parkinson's disease was a highly heterogeneous disease. However, the iron deposition in the paleostriatum continued to progress throughout the course of the disease and accelerated in the late stage of the disease. Dynamic observation of the iron deposition measured by QSM in these areas can be used to monitor the progress of the disease.

Since Lehermitt found the increase of brain iron content in patients with PD in 1924, a number of subsequent postmortem and *in vivo* studies have supported this discovery (2). High concentration of iron (especially ferrous ions) would activate the oxidative stress reaction, enhancing the formation of free radicals (Fenton Reaction) and leading to cell death. In addition, mounting evidence revealed that excessive iron content in the brain was closely related to the main pathological basis of PD: the death of dopaminergic neuronal in the substantia nigra and aggregation of misfolded α-synuclein in Lewy bodies (4, 5).

MRI-based evaluation is now the most widely used for the investigation of cranial iron content *in vivo* in PD patients owing to its advantages of high spatial resolution, non-invasiveness, multi-sequences, and high repeatability (26, 27). In the past decade, conventional MRI techniques for estimating the iron content including T2, T2^*^, and R2' and SWI phase values and results from previous studies proved the excessive iron content in SN (7). Nevertheless, measurements of cranial iron content in these studies were not accurate enough, which may be partly attributed to the various unwanted distortions created by the soft-tissue contrasts in brain such as water and white matter (28). So, in order to solve this limitation, Yi Wang's research team at Cornell University in the United States has proposed quantitative magnetic susceptibility (QSM) *via* a series of complex post-processing, which compensates for the non-locality of magnetic field distribution and provides quantitative measures of magnetic susceptibility by combining magnitude and phase data based on susceptibility weighted imaging (SWI) (29). Currently, the emerging QSM technique is validated to be the most accurate method to quantitatively calculate the cranial iron content and linearly correlated with the iron concentration detected by postmortem MRI and spectrometry (12–14). A series of recent studies used the voxel-wise analysis of QSM to study the whole-brain pattern of iron deposition of PD patients (10, 30). At present, there have been mounting studies on detecting the brain iron *in vivo* in PD and other parkinsonian disorders using the QSM technique (27, 31), which also supports the fact that the conclusions of our research based on the QSM method are accurate and reliable.

We noted that the QSM values in SN and GP in ESP were elevated compared with that of control, although this tendency did not reach statistical significance, while in the ASP patients, the QSM values in SN and GP were significantly higher than that of the control group and ESP patients. In the early stage of PD, the increase in iron content within SN and GP may not be obvious, while in the advanced stage of PD, iron accumulation significantly increased with the progression of PD severity. This conclusion of excessive and progressive iron deposition in SN and GP was consistent with several previous investigations (32–35). Previous studies and our studies have confirmed that patients with PD exhibited significantly higher magnetic susceptibility values, especially in those who are in advanced disease severity stage. There is substantial evidence for a gradual increase of iron deposition in the SN and GP with disease progression, which is related to motor symptoms, so it can be used to observe the progress of the disease. We believe that SN and GP share interconnected neural circuits, which is the anatomical basis of this phenomenon. Previous studies have shown a wide range of neural circuits and projections between SN and GP, and these structures also played a role in movement control and regulation (36, 37).

Time-specific characteristics of iron deposition in RN and PT were also confirmed in this study. Although with the emergence of QSM technology, the qualitative analysis of iron content is more and more accurate, many studies have not reached a consensus on the changes in iron content in RN and PT, and many studies have found that the iron content of PT has not increased (25, 35). Our study found that iron deposition in PT and RN is obviously consistent. Subgroup analysis showed that the iron deposition of RN and PT prominently increased in the early stage of PD patients compared to the control group, and the iron content in RN and PT did not continue to increase in the late stage of PD cases. Further ROC analysis also suggested that the measurement of QSM values in RN and PT regions may provide a potential biomarker for the diagnosis of early PD. So, iron deposition in the neostriatum should be paid more attention in the early stage of the disease, even in the preclinical stage, in future research.

The time-specific difference of iron deposition in different brain regions is related to many factors: (1) There are obvious physical boundaries between different anatomic circuits, which make it difficult to influence each other. The RN–PT network can be dissociated from a nigro-centered striatal network despite the anatomic proximity between the RN and SN (38). (2) The time and the degree of pathological changes in different loops were different. A previous study by Dennis et al. (39) found the dopaminergic nerve fibers in putamen to be decreased as early as in the incidental Lewy body disease (iLBD), which was one disorder that would have eventually progressed to PD, while at that time, there was not yet an obvious neuronal loss in the SN. Another postmortem investigation of prodromal PD patients (40) also found the decreased uptake and loss of TH-immunoreactive nerve fibers in the putamen. These results suggested that the dopaminergic deficiency in putamen and nucleus of neostriatum may occur relatively earlier than the SN and GP involvement in the PD process and may lead to early iron accumulation, which supported our hypothesis.

Clinical data from PD cases analyzed by Pearson correlation analysis showed the QSM values in SN and GP to be significantly correlated with H-Y stage, UPDRS-III scores, and HAMD scores. The more iron accumulated in SN, the more advanced condition and the more severe the motor symptoms of PD, which was consistent with most previous reports (25, 32, 34, 41). We speculated that the iron load in the globus pallidus might be similar to the substantia nigra in pathophysiological effects of PD by means of the structure of SNr–GPi. The SNr–GPi complex is considered to be a part of the basal ganglia circuitry, and the dopaminergic SNc projection to the striatum regulates corticostriatal transmission and integrates the motor information by direct and indirect pathways through this motor circuitry. The SNr–GPi complex has been proven to play a pivotal role in the occurrence and progression of the extrapyramidal disease (42).

Our results demonstrated that the depression in PD might be closely relevant to the excessive iron content in SN, and no other correlation was found between regional QSM values with non-motor symptoms. Liu et al. (34) detected the iron deposition in SN by SWI and found that it was related to several non-motor symptoms of PD such as mild cognitive impairment, sleep disorder, but not depression. An et al. (32) reported that the QSM values significantly correlated with HAMA and MADRS (Montgomery Asberg Depression Rating Scale). Thus, the iron content in SN not only may have correlations with motor symptoms but also may be related to some non-motor symptoms such as depression. However, no correlation was found in other non-motor symptoms; the possible reasons are as follows: hyposmia and constipation are attributed to the dysfunction of peripheral nerve and autonomic nerve, which had no close relevance with iron deposition in the central nervous system. Moreover, cognitive dysfunction in PD is often associated with cortex involvement, which is the last affected area according to Braak staging (43). So, in this study, absence of correlative results may be due to the lack of PD patients in advanced stage. Finally, it is also valuable to expand the research to other brain regions in the future, such as amygdala and hippocampus, which are more closely related to the depression/anxiety and cognitive impairment in order to have a deeper understanding of the non-motor symptoms.

## Limitations

There are several limitations in the present study. First, we evaluated the QSM values in the SN or GP as a whole without subdividing these regions (e.g., subdivide the SN into the pars compacta and pars reticulata, and subdivide the GP into an internal segment and an external segment), which might lead to ambiguous and inaccurate conclusions. Second, the QSM values may be interrupted by other factors such as calcium, copper, or lipid, and the QSM technique is incapable of determining the exact iron content directly. Third, all cases in the PD group are not longitudinal assessment and lack of pathological evidence of PD diagnosis. Therefore, longitudinal cohort studies should also be performed to observe the iron deposition of more precise subregions in the brain. Finally, we did not use voxel-wise analysis of QSM to analyze whole brain iron deposition.

## Conclusion

In this study, we reported the time-specific patterns of regional iron deposition in PD. The increased iron deposition of the RN and PT (neostriatum) is mainly accumulated at the early stage of PD, while in the late stage of PD, the iron deposition in these two regions does not seem to continue to increase. The progressive pattern of iron deposition in the SN and GP (paleostriatum) might be throughout the whole course of PD, particularly occurring in the late stage of PD, which is in line with PD progression and the severity of motor symptoms. Moreover, there may be a potential relevance between iron content in the SN and GP with some of non-motor symptoms such as depression. This study provides the evidence of time-specific iron deposition in different regions in PD.

## Data Availability Statement

The original contributions presented in the study are included in the article/supplementary material, further inquiries can be directed to the corresponding author/s.

## Ethics Statement

The studies involving human participants were reviewed and approved by the Ethics Committee of Nanfang Hospital. The patients/participants provided their written informed consent to participate in this study. Written informed consent was obtained from the individual(s) for the publication of any potentially identifiable images or data included in this article.

## Author Contributions

YX, XF, and QW designed the experiments. XF, JX, XChi, and YJ carried out the experiments and collected the data. MP, WS, and XCui processed images. YX measured the experiment data and XF analyzed the data and experimental results. XF and YX wrote the manuscript. All authors contributed to the article and approved the submitted version.

## Conflict of Interest

The authors declare that the research was conducted in the absence of any commercial or financial relationships that could be construed as a potential conflict of interest.

## Publisher's Note

All claims expressed in this article are solely those of the authors and do not necessarily represent those of their affiliated organizations, or those of the publisher, the editors and the reviewers. Any product that may be evaluated in this article, or claim that may be made by its manufacturer, is not guaranteed or endorsed by the publisher.

## Abbreviations

PD: Parkinson's disease
QSM: quantitative susceptibility mapping
SN: substantia nigra
RN: red nucleus
CN: head of the caudate nucleus
GP: globus pallidus
PT: putamen
H-Y stage: Hoehn-Yahr stage
UPDRSIII: Motor scores of Unified PD Rating Scale
ESP: early stage of PD
ASP: advanced stage of PD
NMSS: Non-motor Symptoms Scale
PQSI: Pittsburgh Sleep Quality Index
MMSE: Mini-Mental Status Examination
HAMA: Hamilton Anxiety Scale
HAMD: Hamilton Depression Scale
MNMSS: Main Non-Motor Symptom Score.
